# Downregulation of lung miR-203a-3p expression by high-altitude hypoxia enhances VEGF/Notch signaling

**DOI:** 10.18632/aging.102878

**Published:** 2020-02-29

**Authors:** Wei Cai, Sanli Liu, Ziquan Liu, Shike Hou, Qi Lv, Huanhuan Cui, Xue Wang, Yuxin Zhang, Haojun Fan, Hui Ding

**Affiliations:** 1 School of Disaster Medical Research, Tianjin University, Tianjin 300072, China; 2 Logistics University of Chinese People’s Armed Police Force, Tianjin 300162, China; 3 Health Company, 95985 Troops of PLA, Kaifeng 475000, Henan province, China; 4 Characteristic Medical Center of Chinese People’s Armed Police Force, Tianjin 300162, China; 5 Medical Team of the Third Detachment of Beijing Armed Police Corp, Beijing 100000, China; 6 The Second Hospital Affiliated Shaanxi University of Chinese Medicine, Shaanxi province, Xianyang 710054, China

**Keywords:** hypoxic lung injury, high altitude, miRNA, miR-203a-3p, VEGF/Notch pathway

## Abstract

Hypoxia-related microRNAs (miRNAs) are involved in the pathogenesis of various diseases. Because potential variations in miRNA expression mediated by hypoxic lung injury at high altitude remain incompletely characterized, we used a rat model to investigate the biochemical and miRNA changes induced by high-altitude hypoxia. After 24, 48, or 72 h of hypoxic exposure, expression of VEGF/Notch pathway-related proteins were increased in rat lung tissues. Microarray screening of hypoxic lung samples revealed 57 differentially expressed miRNAs, 19 of which were related to the VEGF/Notch signaling pathway. We verified that the top downregulated miRNA (miR-203a-3p) suppresses VEGF-A translation through direct binding and also indirectly reduces Notch1, VEGFR2, and Hes1 levels, which restricts the angiogenic capacity of pulmonary microvascular endothelial cells in vitro. These findings may aid in the development of new therapeutic strategies for the prevention and treatment of hypoxic lung injury at high altitude.

## INTRODUCTION

Hypoxia is the most critical cause of acute mountain sickness (AMS) [1, 2]. At high-altitude environments (above 2,500 meters) air oxygen levels remain constant, but as the altitude increases the oxygen partial pressure (PaO2) drops [3, 4]. In non-acclimatized individuals, this reduction in PaO2 can cause arterial desaturation or hypoxemia, which restricts the diffusion of oxygen into alveolar capillaries and lung tissue and may lead to life-threatening conditions, i.e. high-altitude pulmonary edema (HAPE) and high-altitude cerebral edema (HACE) [3, 5]. Normally, the alveolar capillary barrier maintains the balance of fluid exchange in the alveoli. Research showed that in high-altitude environments, hypoxia is the main stimulating factor of angiogenesis, a physiological response to oxygen deprivation [6]. Angiogenesis is characterized by sprouting of vascular endothelial cells under the induction of a vascular endothelial growth factor (VEGF) concentration gradient; however, the capillary tube wall of the newly sprouted growth is weak, the endothelial cell basement membrane is incomplete, and cell gaps tend to be large. Final remodeling of newly formed vessels is mediated by a feedback loop involving activation of Notch signaling [7]; insufficient or defective activation of this pathway allows sustained VEGF production, which leads to increased vascular permeability and may precipitate interstitial edema [8]. Our previous human studies revealed several SNPs in the VEGF and other hypoxia-related genes in association with increased AMS susceptibility in Han Chinese individuals, as well as increased basal plasma VEGF levels in subjects affected by AMS [9, 10]. However, the regulation of the VEGF/Notch pathway and its impact on angiogenic events related to hypoxic lung injury at high-altitude are not fully understood.

MicroRNAs (miRNAs) are a class of small non-coding RNAs, 21-25 nt in length, that exert post-transcriptional gene regulation by base-specific pairing to the 3′-untranslated region (3′-UTR) of target mRNAs. Kulshreshtha et al. were the first to employ gene chip technology to screen 27 hypoxia-related miRNAs [11]. Although later investigations further discovered tissue-specific miRNAs closely correlated with angiogenesis [12–14], only a few studies examined the changes in miRNAs expression associated with hypoxic lung injury at high-altitude [15]. Therefore, we established a rat model of hypoxic lung injury at high-altitude and evaluated by miRNA microarray, qRT-PCR, and protein expression assays, the differential regulation of miRNAs and their hypoxia/angiogenesis related gene targets. These studies were complemented by in vitro assays on an hypoxic cell model using pulmonary microvascular endothelial cells (PMVECs). Our data indicates that exposure to high-altitude hypoxia strongly downregulates miR-203a-3p, which bids to VEGF-A and represses its translation, modulating in turn VEGF/Notch signaling activity and lung angiogenesis.

## RESULTS

### Analysis of high-altitude hypoxic lung injury

**Figure 1:**
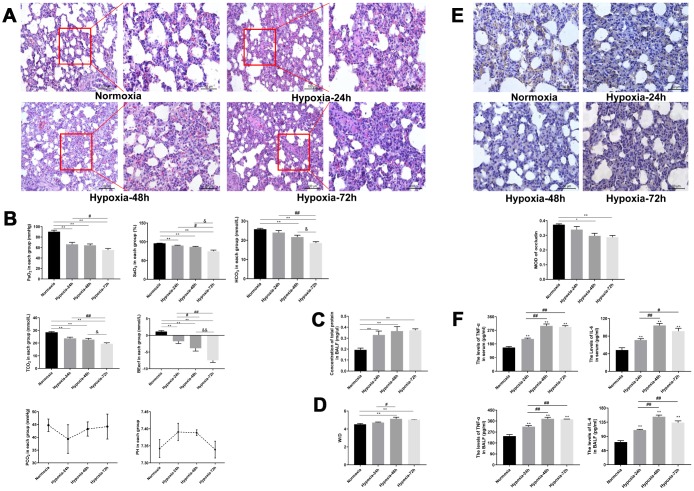
**Dynamic changes in hypoxic lung injury-related indicators.** (**A**) H&E staining of rat lung tissue. Magnifications of the same section (200X and 400X) are shown. (**B**) Analysis of arterial blood gasses. (**C**, **D**) Measurements of total protein concentration in BALF and lung tissue W/D. (**E**) Immunohistochemical staining of occludin in rat lung tissue (400X). (**F**) ELISA analysis of IL-6 and TNF-α levels in serum and BALF. Data are mean ± SEM. ^**^P < 0.01 compared with the normoxic control group; ^#^P < 0.05, ^##^P < 0.01 compared with the 24-h hypoxia group; ^&^P < 0.05, ^&&^P < 0.01 compared with the 48-h hypoxia group.

We evaluated histological, biochemical, and molecular changes in rat’s lung tissue resulting from simulated high-altitude hypoxia lasting 24, 48, or 72 h (see Materials and Methods for experiment details). All the rats showed good tolerance and survived the exposure to simulated hypoxic environment at high altitude. The normoxia group showed essentially no fluid or inflammatory cell exudate in the alveoli and well-preserved alveolar septum structure. In the hypoxia-exposed groups the intrapulmonary structure was damaged, red blood cells were visible in the alveoli and pulmonary septum, and pink fluid exudate was found in the alveoli. The pulmonary artery was congested and the pulmonary septum was significantly widened, showing also infiltrating neutrophils and macrophages (Figure 1A).

**Figure 2:**
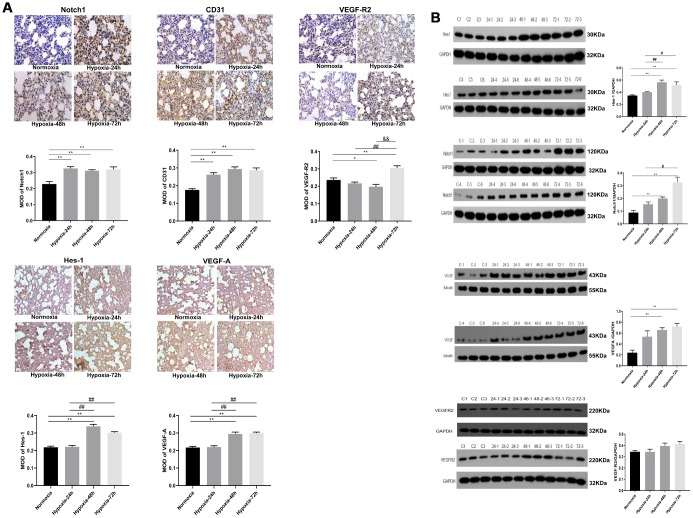
**Expression of VEGF/Notch pathway-related proteins and CD31 in rat lung tissues.** (**A**) Immunohistochemical staining of Notch1, CD31, and VEGFR2 (400X), and VEGF-A and Hes-1 (200X). (**B**) Western blotting analysis of Hes-1, Notch1, VEGF-A and VEGFR2 expression in rat lung tissue. Data are mean ± SEM. ^*^P<0.05, ^**^P<0.01 compared with the normoxic control group; ^#^P<0.05, ^##^P<0.01 compared with the 24-h hypoxia group. ^&&^P<0.01 compared with the 48-h hypoxia group.

As shown in Figure 2B, PaO_2_ and SaO_2_ were markedly reduced in rats exposed to 24, 48, or 72 h of hypoxia (P < 0.01). In turn, significant time-dependent reductions in PaO_2_ and SaO_2_ were observed among the hypoxia groups. We also measured TCO_2_, HCO_3_, and BEecf, i.e. three common indicators of acid-base metabolism. Compared with the normoxia group, TCO_2_ and BEecf were notably reduced in the three hypoxia exposure groups (P < 0.01), whereas HCO_3_ levels were dramatically decreased after 48 and 72 h of hypoxia (P < 0.01). TCO_2_, HCO_3_ and BEecf were all markedly reduced after 72-h hypoxia, compared with the values recorded at shorter treatment times (Figure1B)

Analysis of bronchoalveolar lavage fluid (BALF) showed higher total protein concentration in the three hypoxia exposure groups than in normoxic rats (P < 0.01) (Figure 1C). Also, lung tissue wet/dry ratio (W/D) in the 48- and 72-h hypoxia groups was significantly higher than in normoxic rats (P < 0.01) (Figure 1D). Immunohistochemical analysis showed that lung occludin expression partially decreased after 24 h hypoxia, and was significantly downregulated as hypoxia was prolonged for 48 and 72 h (P < 0.01; Figure 1E). Moreover, ELISA showed that the levels of TNF-α and IL-6 in both serum and BALF increased time-dependently with hypoxia (Figure 1F).

### Hypoxia induces dynamic expression changes in angiogenesis-related proteins in the lung

The effect of hypoxia on the expression of angiogenesis-related proteins in lung tissues was evaluated by immunohistochemistry (IHC) and western blotting (Figure 2). IHC staining showed upregulation of Notch1 and CD31 expression in the three hypoxia groups (P < 0.01); typically, Notch1 signal was strongly positive in the 24-h hypoxia group, while CD31 signal was strongly positive in the 48-h hypoxia group. Compared with the normoxia group, VEGFR2 showed a gradually weakened immunoreactivity in the 24-h and 48-h hypoxia groups, with the lowest expression detected after 48 h of hypoxia (P < 0.05). However, significant VEGFR2 upregulation, compared to the shorter hypoxia treatment groups, could be observed after 72 h of hypoxia (P < 0.01). Taken together, these findings suggest that VEGFR2 expression in alveolar wall epithelial cells might be inhibited by the Notch pathway [16]. Meanwhile, the immunoreactivity of both Hes-1 and VEGF-A increased significantly in the 48-h and 72-h hypoxia groups (P < 0.01) (Figure 2A).

Western blotting Results showed that the expression of Hes-1, Notch1, and VEGF-A was dramatically upregulated in the 48-h and 72-h hypoxia groups compared with the normoxia control group (P < 0.01). Compared with normoxic samples, the expression of VEGFR2 was decreased after 24-h hypoxia, and upregulated after 48 and 72 h of hypoxic exposure, although no significant differences were observed relative to the normoxia group. Compared with the 24-h hypoxia group, the expression of Hes-1 was markedly increased in the 48-h hypoxia group (P < 0.01), while the expression of Hes-1 and Notch1 was further increased after 72 h of hypoxia (P < 0.05) (Figure 2B).

### Screening and validation of hypoxia-sensitive miRNAs

**Figure 3:**
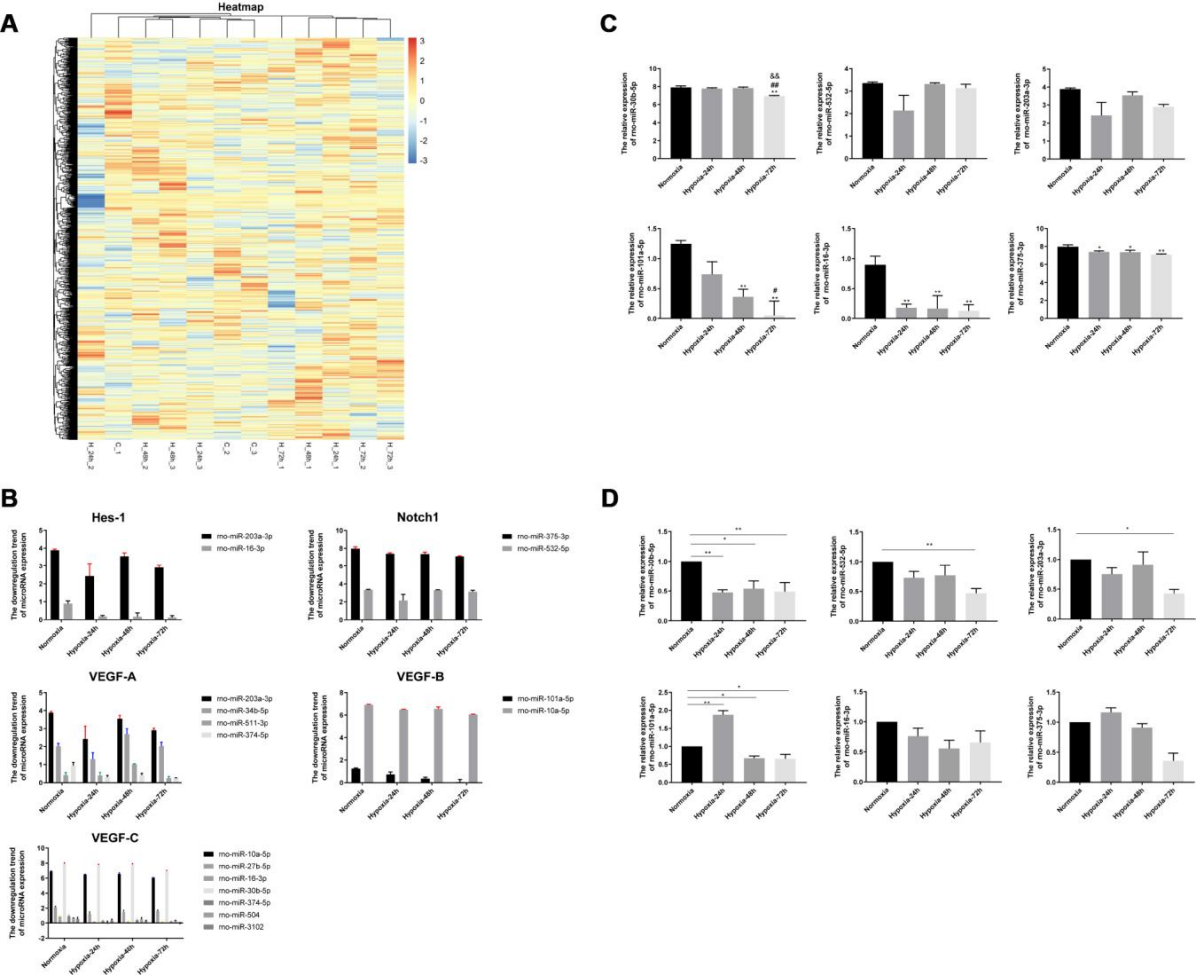
**Screening and validation of miRNAs associated with hypoxic exposure.** (**A**) Heatmap of miRNA microarray data. Unmonitored hierarchical clustering analysis was conducted for differentially expressed genes induced by hypoxia (24,48 and 72h) in the rat lung. A total of 57 miRNAs showed > 1.5 fold change relative to normoxic, control lung tissue samples; blue indicates downregulation. (**B**) Downregulation of miRNAs targeting Hes-1, Notch1, VEGF-A, VEGF-B, and VEGF-C by hypoxia. (**C**) Screening of 6 selected differentially expressed miRNAs associated with the VEGF/Notch pathway. (**D**) Verification of selected miRNAs expression in rat lung by qRT-PCR. Values are expressed as fold change ± SEM relative to control. ^*^P < 0.05, ^**^P < 0.01 compared with the normoxic control group; ^#^P <0.05, ^##^P < 0.01 compared with the 24-h hypoxia group; ^&&^P < 0.01 compared with the 48-h hypoxia group.

A miRNA array chip revealed 57 significant differentially expressed miRNAs between normoxic and hypoxic lung specimens (Figure 3A). Using TargetScan, 19 miRNAs related to the VEGF/Notch pathway, including 6 upregulated and 13 downregulated ones, were identified (Table 1). According to our western blotting and IHC results, the miRNAs related to the VEGF/Notch pathway that were downregulated by hypoxic exposure were screened in our lung samples (Table 2). Figure 3B and 3C show the comparative expression of the miRNAs targeting VEGF, Notch1, and Hes1 in the normoxia and hypoxia groups. Compared with the normoxia group, significant downregulation of rno-miR-30b-5p (P < 0.01), rno-miR-101a-5p (P < 0.01), rno-miR-16-3p (P < 0.01), and rno-miR-375-3p (P < 0.05; P < 0.01) was detected in the different hypoxia groups: compared with 24-h hypoxia, significant downregulation of rno-miR-30b-5p (P < 0.01) and rno -miR-101a-5p (P < 0.05) was detected in the 72-h hypoxia group. Compared with 48-h hypoxia, further downregulation of rno-miR-30b-5p (P < 0.01) was detected in the 72-h hypoxia group. Finally, rno-miR-203a-3p, rno-miR-532-5p, rno-miR-101a-5p, rno-miR-30b-5p, rno-miR-375-3p, and rno-miR-16-3p were selected for quantitative real time polymerase chain reaction (qRT-PCR) validation (Table 3). As shown in Figure 3D, qRT-PCR results suggested that expression variations in rno-miR-203a-3p, rno-miR-532-5p, and rno-miR-30b-5 under hypoxia were consistent with those detected in the miRNA array. The relative expression of rno-miR-101a-5p in the 48- and 72-h hypoxia groups was dramatically downregulated compared with that in the normoxia group (P<0.05). Although the relative expression of rno-miR-375-3p and rno-miR-16-3p in the three hypoxia exposure groups was also decreased in relation to normoxic samples, the differences were not statistically significant.

**Table 1:** VEGF/Notch pathway-related miRNAs differentially regulated by hypoxia in rat. lung tissue.

	**MicroRNA**	**Sequence (5’ to 3’)**	**Regulation**	**Fold Change**	**P-Value**
1	rno-miR-324-5p	CGCAUCCCCUAGGGCAUUGGUGU	Up	9.894773387	0.00000653
2	rno-miR-344g	AGUCAGGCUCCUGGCAGGAGUC	Up	3.196884599	0.015123517
3	rno-miR-203a-3p	GUGAAAUGUUUAGGACCACUAG	Down	2.751083636	0.021135344
4	rno-miR-34b-5p	AGGCAGUGUAAUUAGCUGAUUGU	Down	2.60870414	0.007279015
5	rno-miR-532-5p	CAUGCCUUGAGUGUAGGACUGU	Down	2.329467173	0.031149458
6	rno-miR-101a-5p	UCAGUUAUCACAGUGCUGAUGC	Down	2.281527432	0.000709237
7	rno-miR-30b-5p	UGUAAACAUCCUACACUCAGCU	Down	1.940820463	0.000182969
8	rno-miR-27b-5p	AGAGCUUAGCUGAUUGGUGAACAG	Down	1.844632387	0.033310866
9	rno-miR-375-3p	UUUGUUCGUUCGGCUCGCGUGA	Down	1.823444977	0.008110155
10	rno-miR-10a-5p	UACCCUGUAGAUCCGAAUUUGUG	Down	1.798341071	0.000526176
11	rno-miR-503-5p	UAGCAGCGGGAACAGUACUGCAG	Up	1.74916534	0.008594744
12	rno-miR-511-3p	AAUGUGUAGCAAAAGACAGGA	Down	1.705269784	0.002823832
13	rno-miR-16-3p	ACCAAUAUUAUUGUGCUGCUU	Down	1.697407943	0.003387312
14	rno-miR-374-5p	AUAUAAUACAACCUGCUAAGUG	Down	1.609560345	0.00320276
15	rno-miR-146b-5p	UGAGAACUGAAUUCCAUAGGCUGU	Up	1.551144762	0.014695569
16	rno-miR-504	AGACCCUGGUCUGCACUCUGUC	Down	1.551144762	0.02847243
17	rno-miR-3102	CUCUACUCCCUGCCCCAGCCA	Down	1.547564994	0.033318598
18	rno-miR-376b-3p	AUCAUAGAGGAACAUCCACUU	Up	1.543993487	0.013411178
19	rno-miR-212-3p	UAACAGUCUCCAGUCACGGCCA	Up	1.515716567	0.023319339

Fold change (FC) value represents the maximum value of tatio between the expression level of one of the hypoxic groups at 24h, 48h and 72h and that of the normoxic control group.

**Table 2:** MicroRNAs targeting VEGF/Notch pathway-related mRNAs.

**Targeted mRNA**	**MicroRNA**	**Sequence (5' to 3')**	**Regulation**	**Fold Change**	**P-Value**
Hes-1	rno-miR-203a-3p	GUGAAAUGUUUAGGACCACUAG	Down	2.751083636	0.021135344
	rno-miR-16-3p	ACCAAUAUUAUUGUGCUGCUU	Down	1.697407943	0.003387312
Notch1	rno-miR-375-3p	UUUGUUCGUUCGGCUCGCGUGA	Down	1.823444977	0.008110155
	rno-miR-532-5p	CAUGCCUUGAGUGUAGGACUGU	Down	2.329467173	0.031149458
VEGF-A	rno-miR-203a-3p	GUGAAAUGUUUAGGACCACUAG	Down	2.751083636	0.021135344
	rno-miR-34b-5p	AGGCAGUGUAAUUAGCUGAUUGU	Down	2.60870414	0.007279015
	rno-miR-511-3p	AAUGUGUAGCAAAAGACAGGA	Down	1.705269784	0.002823832
	rno-miR-374-5p	AUAUAAUACAACCUGCUAAGUG	Down	1.609560345	0.00320276
VEGF-B	rno-miR-101a-5p	UCAGUUAUCACAGUGCUGAUGC	Down	2.281527432	0.000709237
	rno-miR-10a-5p	UACCCUGUAGAUCCGAAUUUGUG	Down	1.798341071	0.000526176
VEGF-C	rno-miR-10a-5p	UACCCUGUAGAUCCGAAUUUGUG	Down	1.798341071	0.000526176
	rno-miR-27b-5p	AGAGCUUAGCUGAUUGGUGAACAG	Down	1.844632387	0.033310866
	rno-miR-16-3p	ACCAAUAUUAUUGUGCUGCUU	Down	1.697407943	0.003387312
	rno-miR-30b-5p	UGUAAACAUCCUACACUCAGCU	Down	1.940820463	0.000182969
	rno-miR-374-5p	AUAUAAUACAACCUGCUAAGUG	Down	1.609560345	0.00320276
	rno-miR-504	AGACCCUGGUCUGCACUCUGUC	Down	1.551144762	0.02847243
	rno-miR-3102	CUCUACUCCCUGCCCCAGCCA	Down	1.547564994	0.033318598

Fold change (FC) value represents the maximum value of tatio between the expression level of one of the hypoxic groups at 24h, 48h and 72h and that of the normoxic control group.

**Table 3:** List of 6 mRNAs related to the VEGF/Notch pathway by prediction of miRNA microarrays.

	**MicroRNA**	**Gene**	**Gene discription**	**Sequence (5′to3′)**
1	rno-miR-203a-3p	Vegfa	vascular endothelial growth factor A	GUGAAAUGUUUAGGACCACUAG
		Hes1	hairy and enhancer of split 1, (Drosophila)	GUGAAAUGUUUAGGACCACUAG
2	rno-miR-532-5p	Notch1	notch 1	CAUGCCUUGAGUGUAGGACUGU
3	rno-miR-375-3p	Notch1	notch 1	UUUGUUCGUUCGGCUCGCGUGA
4	rno-miR-16-3p	Hes1	hairy and enhancer of split 1, (Drosophila)	ACCAAUAUUAUUGUGCUGCUU
		Vegfc	vascular endothelial growth factor C	ACCAAUAUUAUUGUGCUGCUU
5	rno-miR-101a-5p	Vegfb	vascular endothelial growth factor B	UCAGUUAUCACAGUGCUGAUGC
6	rno-miR-30b-5p	Vegfc	vascular endothelial growth factor C	UGUAAACAUCCUACACUCAGCU

### Hypoxia regulates the expression of angiogenesis-related miRNAs and their gene targets in PMVECs

**Figure 4:**
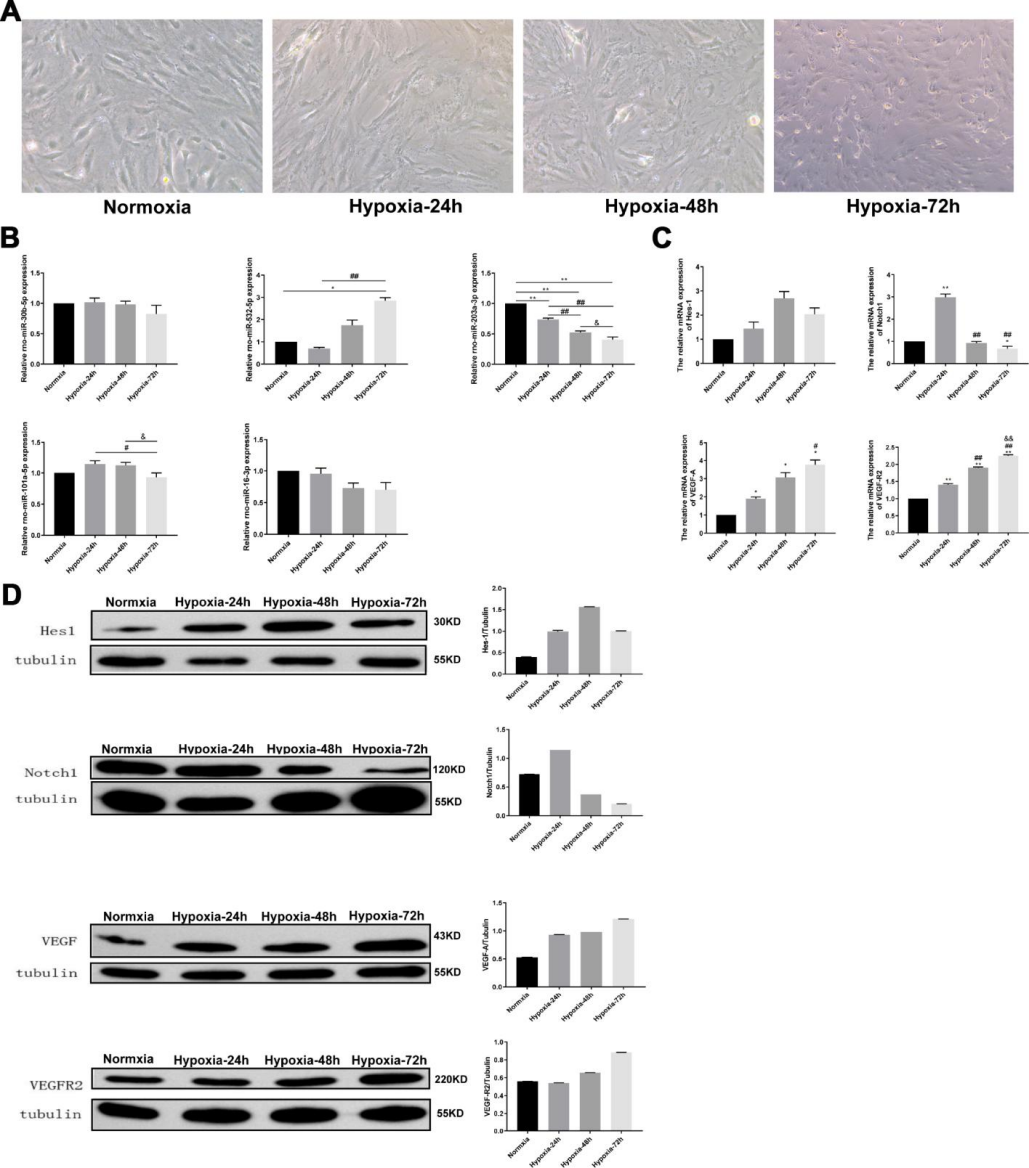
**Analysis of hypoxia-induced changes in VEGF/Notch signaling effector molecules and related miRNAs in PMVECs.** (**A**) PMVECs were cultured under hypoxia for 0, 24, 48, or 72 h. With prolongation of hypoxic exposure the cells lost their original morphology and cell population density decreased (200X). (**B**) Relative expression of miR-30b-5p, miR-532-5p, miR-203a-3p, miR-101a-5p, and miR-16-3p assessed by qRT-PCR. (**C**) Relative expression of Hes-1, Notch1, VEGF-A, and VEGFR2 mRNA measured by qRT-PCR. (**D**) Hes-1, Notch1, VEGF-A, and VEGFR2 expression assessed by western blotting. Data are mean ± SEM. ^*^P < 0.05, ^**^P < 0.01 compared with the normoxic control group; ^#^P < 0.05, ^##^P < 0.01 compared with the 24-h hypoxia group; ^&^P < 0.05, ^&&^P < 0.01 compared with the 48-h hypoxia group.

After culturing PMVECs in normoxic or hypoxic (2% O_2_) conditions for 24, 48, or 72 h, morphological inspection was carried out under a high power microscope. Control cells grown under normoxia showed typical growth kinetics with long, spindle-shaped morphology. With increased hypoxia times, however, a negative impact on cell growth was evident, manifested by disordered distribution, reduced cell numbers, and loss of inherent morphological characteristics (Figure 4A). Next, qRT-PCR was used to evaluate in PMVECs the expression of the hypoxia-related miRNAs identified in our animal hypoxia model by microarray assay, as well as their predicted target mRNAs. A downward expression trend of rno-miR-30b-5p, rno-miR-203a-3p, rno-miR-101a-5p, and rno-miR-16-3p was found in PMVECs with oxygen-deprived cultures at 24, 48, and 72 h. Thus, rno-miR-203a-3p expression showed a significant, continuous decline. In contrast, and contrary to the animal model results, the expression of rno-miR-532-5p showed a significant, continuous upward trend (Figure 4B). Figure 4C shows the expression of predicted target mRNAs for the above miRNAs in cultured PMVECs. The expression of Notch1 mRNA showed an initial significant increase during hypoxia incubation, and decreased thereafter. The other three mRNAs, i.e. Hes1, VEGF-A, and VEGFR2, showed an upward expression trend during hypoxia, especially evident for VEGF-A and VEGFR2 transcripts.

In line with these results, western blotting analyses showed transient upregulation of Notch1, and gradual upregulation of Hes-1, VEGF-A, and VEGFR2 expression during prolonged hypoxia (Figure 4D). Based on these results, we selected rno-miR-203a-3p and its predicted target, VEGF-A, to study the effects of this interaction on the VEGF/Notch pathway.

### miR-203a-3p levels modulate the expression of hypoxia-related angiogenesis markers in PMVECs

**Figure 5:**
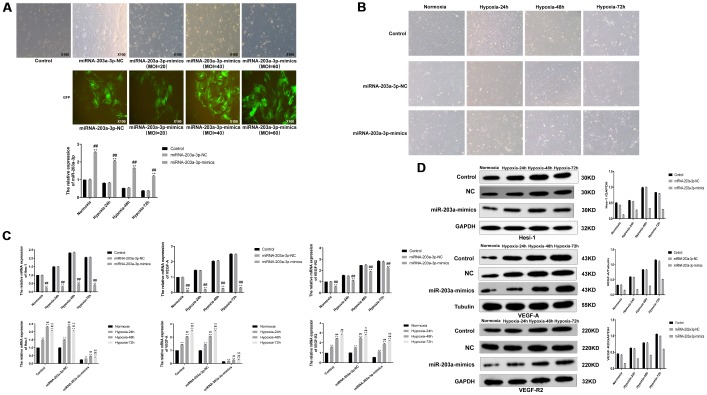
**miR-203a-3p mimics expression inhibits the expression of VEGF-A and downstream genes.** (**A**) Strong GFP fluorescence in PMVECs transduced with miR-203a-3p mimics indicated significantly increased expression of miR-203a-3p, compared with the corresponding control (no transduction) (P < 0.01) (100X). (**B**) Morphological changes in PMVECs transduced with lentiviral vectors encoding miR-203a-3p-NC (negative miRNA-203a-3p mimics control) or miR-203a-mimics (100X). (**C**) Results of qRT-PCR analysis showing decreased expression of Hes-1, VEGF-A, and VEGFR2 in miR-203a-mimics-transduced PMVECs (P < 0.01). (**D**) Western blotting results showing reduction on Hes-1, VEGF-A and VEGFR2 expression in PMVECs transduced with miR-203a-3p-mimics. Data are mean ± SEM. ^**^P < 0.01 compared with the control or normoxic control groups; ^##^P < 0.01 compared with miR-203a-3p-NC or 24-h hypoxia group; ^&^P < 0.05, ^&&^P < 0.01 compared with the 48-h hypoxia group.

When cultured PMVECs reached ~50% confluence, the lentiviral constructs HBLV-GFP-PURO and HBLV-miR-203a-GFP-PURO were introduced at virus titers of 2×108TU/ml for MOI = 10, 40, and 60. Cells were next divided into a control (no transduction) group, a miR-203a-3p-NC (negative miR-203a-3p mimics control) group, and a miR-203a-3p-mimics group. Infection efficiency was determined 72 h later by GFP fluorescence (Figure 5A). Only transduced cells showed GFP activity, while control cells did not. After culturing the above cell groups under normoxic or hypoxic conditions, transduction efficiency was further evaluated by qRT-PCR (Figure 5A). Compared with the control and the miR-203a-3p-NC groups, the expression of miRNA-203a-3p in the mimics group was significantly increased in the normoxia and hypoxia (0, 24, 48, and 72 h). Morphological inspection revealed that cells expressing miRNA-203a-3p-mimics showed a slightly disorganized growth and impaired morphology compared with cells in the control and miR-203a-3p-NC groups (Figure 5B).

To evaluate the impact of miR-203a-3p mimics on hypoxia-related angiogenic signaling, we used qRT-PCR and western blotting to detect the expression of VEGF-A, VEGFR2, and Hes-1 in control (no lentivirus-transduced PMVECs) and lentivirus-transduced PMVECs grown under normoxia or hypoxia. As shown in the Figure 5C, the expression of VEGF-A, VEGFR2, and Hes-1 mRNA was significantly decreased after forced expression of miRNA-203a-3p-mimics under both normoxia and hypoxia (0, 24, 48, and 72 h). Meanwhile, we compared the expression of VEGF-A, VEGF-R2, and Hes-1 in control, miR-203a-3p-NC, and miR-203a-3p-mimics cells after cultivating 0, 24, 48, and 72 h. Compared with the normoxic control group, VEGF-A and VEGFR2 mRNA levels showed a gradual upward trend in cells after cultivating 0, 24, 48, and 72 h. The expression of Hes-1 mRNA was significantly increased compared with the normoxic control group following 24 and 48 h of hypoxia, but decreased significantly after 72 h in cells transduced with miR-203a-3p-mimics.

Protein expression levels for VEGF-A, VEGFR2, and Hes-1 are shown in Figure 5D. VEGF-A and VEGF-R2 showed a gradual upward expression trend in control, miR-203a-3p-NC, and miRNA-203a-3p-mimics cells after cultivating 0, 24, 48, and 72 h. Hes-1 showed a gradual upward expression trend in control, miRNA-203a-3p-NC, and miR-203a-3p-mimics cells after cultivating 0, 24 and 48h, and then downward after 72h. Meanwhile, the observed expression in VEGF-A, VEGFR2, and Hes-1 induced by hypoxia was downregulation after induced expression of miR-203a-3p mimics.

### miR-203a-3p mimics expression promotes apoptosis, impairs angiogenesis, and stimulates pro-inflammatory cytokine expression in PMVECs

**Figure 6:**
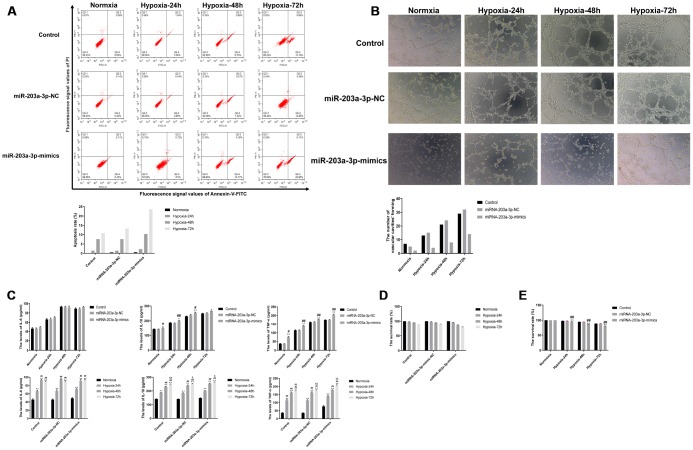
**miR-203a-3p mimics expression decreases survival and angiogenic activity and induces a pro-inflammatory response in PMVECs.** (**A**) Apoptosis assay results showing increased apoptosis rate in PMVECs expressing miR-203a-3p mimics. (**B**) In vitro angiogenesis assay results. PMVECs transduced with miR-203a-3p mimics showed weak angiogenic ability, which improved however with prolonged hypoxic incubation time. (**C**) ELISA assay results showing increased IL-6, IL-10, and TNF-α secretion in PMVECs transfected with miR-203a-3p. (**D**, **E**) CCK8 assay results indicating decreased survival rate in PMVECs transfected with miR-203a-3p mimics. Data are mean ± SEM. ^*^P < 0.05, ^**^P < 0.01 compared with the control (no transduction) or normoxic control groups; ^#^P < 0.05, ^##^P < 0.01 compared with the miR-203a-3p-NC (negative miRNA-203a-3p mimics control) or 24-h hypoxia groups; ^&^P < 0.05, ^&&^P < 0.01 compared with the 48-h hypoxia group.

To study the effect of miR-203a-3p expression on the survival of PMVECs, apoptosis was detected by flow cytometry after labeling cells with annexin V-FITC and PI. Results showed that the apoptosis rate increased gradually with hypoxia incubation time in all cell groups, and was obviously enhanced in those expressing miR-203-3p mimics. As seen in Figure 6A, both early and late apoptotic events were detected in the latter group.

On the other hand, in vitro Matrigel angiogenesis experiments indicated a stimulatory effect of hypoxia on angiogenesis in the three PMVECs groups. However, the angiogenic ability of PMVECs was reduced after transfection with miR-203a-3p-mimics (Figure 6B).

Because pro-inflammatory cytokine production may affect cell proliferation and growth [17, 18], we assessed the effect of miR-203a-3p mimics expression on the secretion of TNF-α, IL-6, and IL-10 by cultured PMVECs. We found that under both normoxia and hypoxia, cell supernatant levels of TNF-α, IL-6, and IL-10 tended to increase after forced miR-203a mimics expression, compared with control (no transduction) and miR-203a-3p-NC-transduced cells. However, with prolonged hypoxia (72 h), TNF-α, IL-6, and IL-10 levels also increased significantly in the remaining cell groups (Figure 6C). These data are consistent with the above described cytokine measurements in serum and BALF, and confirm that prolonged hypoxia exposure stimulates the release of pro-inflammatory factors by pulmonary endothelial cells. We finally tested whether miR-203-3p-mimics expression affects cell viability under normoxic and hypoxic conditions. As shown in Figure 6D, cell viability decreased gradually regardless of transduction status under hypoxia (24, 48 and 72 h). However, after inducing the expression of miR-203-3p mimics cell viability decreased more significantly compared to the control and miR-203a-3p-NC groups (P < 0.01) (Figure 6E).

### VEGF-A is a direct target of miR-203a-3p

**Figure 7:**
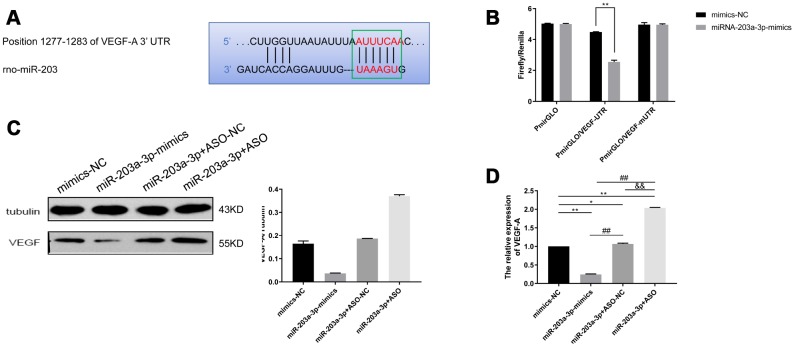
**VEGF-A is a direct target of miR-203a-3p.** (**A**) Sequence information of miR-203 binding sites in the 3′-UTR region of the VEGF-A mRNA. (**B**) Dual luciferase assay indicating decreased fluorescence in PMVECs co-expressing pmirGLO/VEGF-UTR and miR-203-3p mimics. (**C**) Western blotting detection of target genes of miR-203a-3p. Compared with mimics-NC (negative miR-203a-3p mimics control), VEGF expression decreased after transfection with miR-203-mimics, and increased after transfection with miR-203-ASO. (**D**) qRT-PCR detection of target genes of miR-203a-3p. VEGF mRNA levels decreased after transfection with miR-203-mimics, and increased after transfection with miR-203-ASO. Data are mean ± SEM. ^*^P < 0.05, ^**^P < 0.01 compared with mimics-NC; ^##^P < 0.01 compared with miR-203a-3p-mimics; ^&&^P < 0.01 compared with miR-203a-3p-ASO-NC (negative miR-203a-3p-ASO control).

To explore whether VEGF-A is a direct target of miR-203a-3p, we constructed dual-luciferase reporter plasmids containing either the wild-type 3′-UTR sequence of the VEGF-A mRNA (pmirGLO/VEGF-UTR) or a mutant 3′-UTR variant (pmirGLO/VEGF-mUTR) (Figure 7A). These were alternatively co-transfected with vectors containing either miR-203a-3p-mimics or a non-functional miRNA sequence (negative control), and relative luciferase activity was determined for each VEGF construct. There was no change in fluorescence after transfection of pmirGLO/VEGF-mUTR in the presence or absence of miR-203a-3p-mimics, while luciferase fluorescence decreased after transfection of pmirGLO/VEGF-UTR along with the functional miR-203-3p-mimics construct (P < 0.01) (Figure 7B). These data indicated that miR-203-3p has a direct regulatory effect on target VEGF-A mRNA. Next, western blotting and qRT-PCR were used to detect VEGF-A expression in cells transfected with vectors containing either, miR-203a-3p-mimics-NC, miR-203a-3p-mimics, or antisense oligonucleotides (ASO), i.e. miR-203a-3p+ASO-NC (negative miR-203a-3p+ASO control), or miR-203a-3p+ASO. Figure 7C shows that compared with mimics-NC (negative miR-203a-3p mimics control), the expression of VEGF-A was decreased after transduction of miR-203-mimics, and increased after transfection of miR-203-ASO. Accordingly, qRT-PCR analyses showed that compared with mimics-NC, VEGF-A mRNA levels were decreased in cells expressing miR-203a-3p-mimics, and increased instead in those transfected with miR-203a-3p+ASO (Figure7D).

### miR-203a-3p silencing and VEGF-A knockdown effect the growth and survival of PMVECs

**Figure 8:**
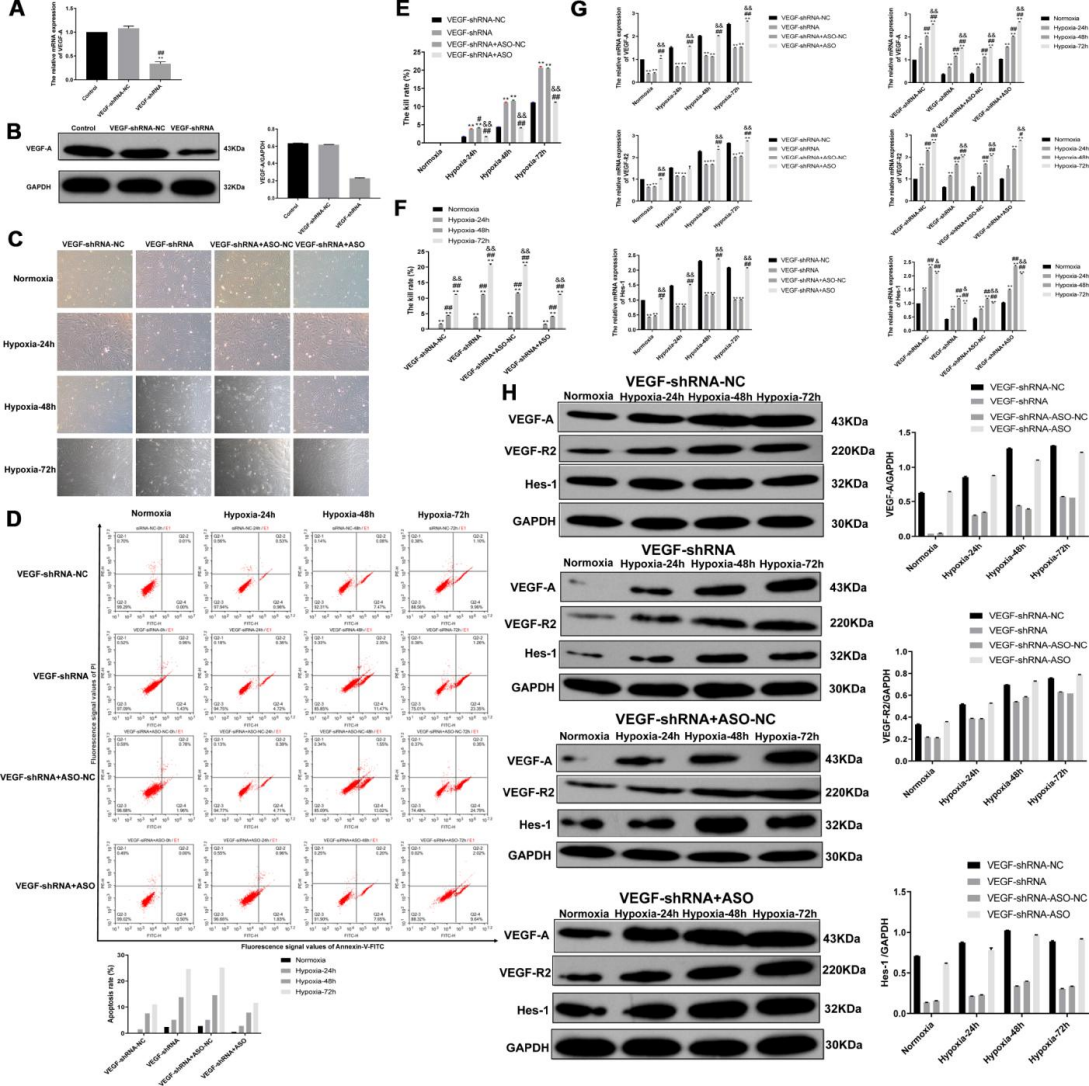
**VEGF-A expression is rescued by miR-203a-3p knockdown.** (**A**) The validation of VEGF-A expression reduction in VEGF-shRNA-transduced cells by qRT-PCR. (**B**) Western blotting data showing reduced expression of VEGF-A in PMVECs transduced with VEGF-shRNA. (**C**) Morphological evaluation in PMVECs transduced with VEGF-shRNA+ASO-NC (negative VEGF-shRNA+ASO control) or VEGF-shRNA+ASO (miR-203a-3p knockdown). Note that inhibition of miR-203a-3p activity reversed morphological impairment caused by VEGF silencing. (**D**) Flow cytometry apoptosis assay indicates increased apoptosis in PMVECs expressing VEGF-shRNA, and reversal of the effect by ASO-mediated miR-203a-3p silencing. (**E**–**F**) CCK-8 assay showing improved viability in PMVECs expressing VEGF-shRNA+ASO, compared with the VEGF-shRNA+ASO-NC group. (**G**) qRT-PCR results showing reduced expression of VEGF-A and its downstream mediators, VEGFR2 and Hes-1, in cells transfected with VEGF-shRNA. The expression of these mRNAs increased instead in cells co-expressing miR-203a-3p-ASO. (**H**) Western blotting results showing protein expression changes consistent with the mRNA data showed in (**G**). Data are mean ± SEM. ^*^P < 0.05, ^**^P < 0.01 compared with VEGF-shRNA negative control (VEGF-shRNA-NC) and normoxia groups; ^#^P < 0.05, ^##^P < 0.01 compared with the VEGF-shRNA or 24-h hypoxia group; ^&^P < 0.05, ^&&^P < 0.01 compared with the VEGF-shRNA+ASO-NC or 48-h hypoxia group.

We further explored the relationship between miR-203a-3p and VEGF-A by transducing PMVECs with lentiviral vectors, i.e. HBLV-VEGF-shRNA-GFP-PURO (VEGF-shRNA) or HBLV-GFP-PURO (VEGF-shRNA negative control) at a MOI of 30, with simultaneous transfection of either ASO-NC (VEGF-shRNA+ASO negative control) or miR-203a-3p-ASO (VEGF-shRNA+ASO). Using qRT-PCR and western blotting, we confirmed that after expression of VEGF-shRNA, VEGF-A mRNA and protein levels declined significantly (P < 0.01) (Figure 8A and 8B). As shown in Figure 8C, after 72 hours the growth pattern of PMVECs in the VEGF-shRNA and VEGF-shRNA+ASO-NC (VEGF-shRNA+ASO negative control) groups was slightly disordered compared with cells in the VEGF-shRNA-NC (VEGF-shRNA negative control) and VEGF-shRNA+ASO group. In contrast, growth characteristics were largely preserved in PMVECs expressing VEGF-shRNA-NC and VEGF-shRNA+ASO.

Apoptosis and growth dynamics were next evaluated by flow cytometry and the CCK-8 assay, respectively, in PMVECs infected with VEGF-shRNA lentivirus. Annexin V-FITC flow cytometry results showed that the apoptotic rate increased gradually with the prolongation of hypoxia, was further increased in cells expressing VEGF-shRNA and VEGF-shRNA+ASO-NC, compared with the VEGF-shRNA-NC group. In contrast, the apoptotic rate of cells in the VEGF- shRNA+ASO group was not different from that of the VEGF-shRNA-NC (Figure 8D). Results of the CCK-8 assay, shown in Figure 8E, showed that during hypoxic incubation for 0, 24, 48, or 72 h the cell death rate increased significantly in the VEGF-shRNA and VEGF-shRNA+ASO-NC groups and compared to cells transduced with VEGF-shRNA-NC (P < 0.01). Again, cell viability decreased with hypoxia time in all groups (P < 0.01) (Figure 8F).

### Inhibition of miR-203a-3p promotes the expression of VEGF-A, VEGFR2, and Hes-1

We analyzed the expression of VEGF-A, VEGFR2, and Hes-1 in cells transduced with VEGF-shRNA or the corresponding negative control and transfected also with ASO-NC or miR-203a-3p-ASO. As shown in Figure 8G, mRNA levels of VEGF-A and VEGFR2 increased gradually with hypoxia (24, 48 and 72 h) in the four groups (VEGF-shRNA-NC, VEGF-shRNA, VEGF-shRNA+ASO-NC and VEGF-shRNA+ASO) of PMVECs and mRNA levels of Hes-1 showed a decrease after 72 h. Meanwhile, VEGF-A, VEGFR2, Hes-1 mRNA expression in the VEGF-shRNA and VEGF-shRNA+ASO-NC groups was significantly lower than in the VEGF-shRNA-NC group. In contrast, VEGF-A, VEGFR2, Hes-1 mRNA expression in the VEGF-shRNA+ASO group was mostly increased compared with the VEGF-shRNA-NC, the VEGF-shRNA or the VEGF-shRNA+ASO-NC groups. Meanwhile, in line with western blotting results confirmed that VEGF-A and VEGF-R2 expression increased with hypoxia (24, 48 and 72 h) in the four cell groups, with a slight decrease in Hes-1 levels observed after 72 h (Figure 8H).

## DISCUSSION

High-altitude hypoxic exposure induces increased IL-6 and TNF-α levels in the body, which cause persistent damage and remodeling of the alveolar capillary barrier, affect permeability and gas exchange, and ultimately lead to pulmonary dysfunction [19–21]. Studies have found that the expression of occludin in a rat model of high-altitude hypoxic lung injury is related to the stability of the alveolar-capillary barrier; as the expression of occludin increases, so does the stability of the alveolar-capillary barrier [22]. In this study, an experimental chamber was used to study the effects of a simulated a hypoxic environment at an altitude of 6,000 m on lung function and expression of miRNA and VEGF/Notch signaling mediators in rats. After hypoxic exposure for 24, 48, and 72 h, arterial blood gas analyses revealed a sustained PO2 decrease, a transient decrease followed by an increase in PCO2, and a reduction in BEecf levels. This indicated an initial hyperventilation response, that gave place to hypoventilation as hypoxia was prolonged [23, 24]. Studies have shown that the decrease in SaO2, also observed in our experiment, is associated with alveolar and interstitial edema, which leads to limited oxygen diffusion [23]. Meanwhile, the increase in TNF-α and IL-6 levels in serum and BALF, as well as the marked increase in W/D weight and total BALF protein content induced by hypoxia were indicative of the occurrence of pulmonary edema and increased pulmonary vascular permeability [25, 26]. Moreover, pathological inspection further suggested the presence of fluid exudation and hemorrhage in the alveoli, along with widening of lung septa and inflammatory cell infiltration. On the other hand, occludin expression was downregulated with the extension of the hypoxia modeling time, confirming along with the above data that simulated plateau hypoxia exposure causes lung tissue damage in rats.

In recent years, a negative feedback loop has been confirmed between the VEGF and Notch pathways during the vascular endothelial remodeling process. Specifically, endothelial tip cells guide the sprouting of new vessels in a process facilitated by VEGF production and stimulated in turn by hypoxia. However, high VEGF induction gradients may result in overproduction of tip cells, resulting in excessive vessel sprouting and formation of highly dense, immature vascular networks [27]. Characteristically, VEGF acts upstream of Notch, which binds and activates VEGFR2 in tip cells, thereby activating the DLL4 promoter of the Notch receptor. Subsequently, DLL4 binds to Notch1/4 and activates downstream targets, including Hes-1, which promotes the production of bHLH transcription factors which further regulate gene expression to ultimately inhibit the formation of tip cells [16, 27, 28]. Thus, the Notch pathway is an important regulator of vascular remodeling by stimulating vessel pruning and shaping the mature vascular network. When the Notch pathway is inhibited, stalk/tip cell specification programs are disrupted, resulting in excessive numbers of tip cells and vascular dysplasia [29, 30]. In this study, we found that the expression of the VEGF/Notch pathway-associated proteins VEGF-A, VEGFR2, Notch1, and Hes-1 in lung tissue was stimulated, along with the expression of the pulmonary microvascular endothelial marker CD31, with prolonged hypoxic exposure times. In contrast, hypoxia caused a decrease in occludin expression in the lung. Subsequent experiments in vitro showed that hypoxia impaired growth dynamics in PMVECs, and promoted the expression of VEGF-A, VEGFR2, Notch1, and Hes-1. These data indicate that hypoxic stimulation activates the VEGF/Notch pathway, which induces angiogenic proliferation of pulmonary endothelial cells.

Mounting evidence supports important roles for miRNAs as regulators of gene expression by suppressing mRNA translation, promoting mRNA degradation, or both [28]. Alam et al. were the first to investigate the role of miRNAs in the pathophysiology of hypoxia at high altitude [15]. They found several miRNAs that were differentially expressed during hypoxia and showed potential involvement in pathophysiological processes closely related to high-altitude pulmonary edema, such as inhibition of ion channels and induction of pulmonary arterial hypertension, as well as promotion of angiogenesis and oxidative stress disorder [15]. Our microarray study revealed that hypoxia exposure induced differential expression of 57 miRNAs in the rat lung. Of these, we focused on 6 downregulated ones related to the Notch/VEGF pathway according to the TargetScan database. We validated the expression of these 6 miRNAs by qRT-PCR and confirmed in in vitro studies in PMVECs that miR-203a-3p, the most significantly downregulated miRNA, was a binding partner of VEGF-A. Importantly, cell culture data showed that miR-203a-3p expression decreased gradually with hypoxic exposure, thus confirming the results of miRNA microarray and qRT-PCR analyses in our animal model.

Hypoxia can induce the expression of vascular growth factors such as VEGF-A, which is dysregulated in some lung diseases. In various types of lung cancer, the HIF-1α/VEGF axis is activated in the hypoxic tumor microenvironment, which promotes angiogenesis and maintains cancer growth [31–33]. In chronic obstructive pulmonary disease (COPD), angiogenesis caused by hypoxia causes irreversible damage to pulmonary blood vessels and thickening of the vascular muscle layer, a condition that may develop into life-threatening pulmonary hypertension [34]. It has been shown that hypoxia modulates the expression of miRNAs involved in angiogenesis [31]. A recent study found that miR-203a-3p is downregulated in non-small cell lung cancer (NSCLC), and silencing of lncRNA LINC00342, which targets miR-203a-3p, inhibits the growth and invasion of cultured NSCLC cells [35]. Our dual luciferase assay results demonstrated direct targeting of VEGF-A by miR-203a-3p, and this was supported by the observation that forced expression of miR-203a-3p mimics inhibited the transcription of the VEGF-A gene and its translation into protein. Thus, we propose that downregulation of miR-203a-3p during hypoxia promotes VEGF-A-mediated angiogenesis in lung, which has clear implications for physiological and pathophysiological processes. Interestingly, we found that ASO-mediated miR-203a inhibition rescued VEGF expression in shRNA-transduced PMVECs. In addition, we showed that miR-203a-3p mimics expression led to increased apoptosis and pro-inflammatory cytokine expression in PMVECs subjected to hypoxia. The results suggested that miR-203a-3p is suggested in recent studies to be associated with apoptosis, which is a kind of apoptosis-related miRNA [36–38].

Previous studies have shown that in cancer cells, miR-203a-3p can directly or indirectly target VEGF-A to regulate tumor angiogenesis, which led to consider miR-203a-3p as a tumor suppressor gene [39]. Recent studies have found that in oxygen-induced rat retinopathy models and high-glucose-induced endothelial-mesenchymal transition of human retinal microvascular endothelial cells, upregulation of miR-203a-3p decreases VEGF-A expression and inhibits pathological retinal angiogenesis [40, 41]. Based on these data and our own results, we conclude that miR-203a-3p negatively regulates VEGF-A and impacts the expression of key downstream effector molecules, regulating pulmonary angiogenesis through the VEGF/Notch pathway.

**Figure 9:**
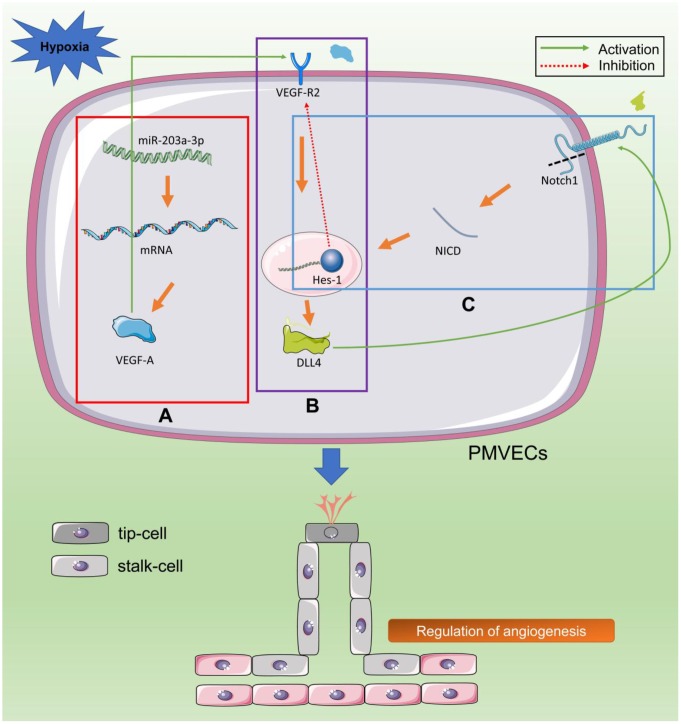
**Hypoxia stimulates VEGF/Notch signaling by downregulating miR-203a-3p expression.** (**A**) Hypoxia upregulates the expression of VEGF-A and downregulates the expression of its negative regulator miR-203a-3p (red box). (**B**) VEGF-A binds and activates VEGFR2 in tip cells, leading to the activation of the Dll4 promoter (purple box). (**C**) Dll4 activates Notch1 receptor in neighboring stem cells. Gamma secretase (GS) cleaves the Notch1 receptor intracellular domain (NICD), which is transferred to the nucleus to enhance the expression of transcription factors (TFs) such as Hes-1. These TFs inhibit the expression of VEGFR2 in stem cells and promote the expression of downstream genes that regulate budding, proliferation, and differentiation of PMVECs (blue box).

In summary, our rat model of hypoxic lung injury at high altitude, along with the present in vitro studies on PMVECs, suggest that downregulation of miR-203a-3p during hypoxia exposure promotes VEGF-A expression and activates the VEGF/Notch pathway, leading to angiogenic proliferation of pulmonary endothelial cells. With prolonged hypoxic exposure, excessive angiogenesis leads to increased permeability of the alveolar capillary barrier, which triggers symptoms of pulmonary edema (Figure 9). Thus, our study provides new insights into the pathogenesis of hypoxic lung injury at high altitude and suggests that novel therapies targeting the miR-203a-3p-VEGF-A interaction may. prove useful to prevent or treat pulmonary dysfunction triggered by hypoxia, including conditions such as AMS, HAPE, and HACE. However, further studies are needed to clarify the molecular events controlling miR-203a-3p expression under different oxygen concentrations

## MATERIALS AND METHODS

### Model of hypoxic lung injury at high altitude

Specific pathogen-free (SPF), adult male Sprague Dawley (SD) rats (180-200 g) were purchased from the Animal Center of the Academy of Military Medical Sciences (production license number: SCXK (Army) 2014-0013). The rats received humane care according to the Guide for the Care and Use of Laboratory Animals from the National Institute of Health (NIH). The study protocol was approved by the Laboratory Animal Ethics Committee of Chinese People’s Armed Police Force (PAP) Medical Center. Animals were pre-conditioned to the indoor environment at 25°C and 60% humidity for two days.

### Animal treatment and sampling

A total of 32 male SD rats were randomly divided into four groups, i.e. group 1: Normoxia (no treatment; n = 8), and groups 2, 3, and 4: hypoxia exposure (continuous exposure to a low temperature, low pressure, hypoxic environment for 24, 48, or 72 h, respectively; n = 8 for each group). After treatment completion, 5 ml of blood was collected from the abdominal aorta immediately after anesthesia with 2% sodium pentobarbital (2 ml/0.1 kg body weight) to carry out arterial blood gas analyses. In the meantime, the right principal bronchus was ligated, the left lung was lavaged twice with 4.5 ml normal saline, and ~4 ml of BALF was aspirated after separating the right lung. The upper lung lobe was used to measure tissue W/D, the right middle lobe was fixed with 4% paraformaldehyde for hematoxylin-eosin (H&E) staining and immunohistochemistry, and the right lower lobe was preserved at -80°C for molecular analyses.

### Experimental chamber

**Figure 10:**
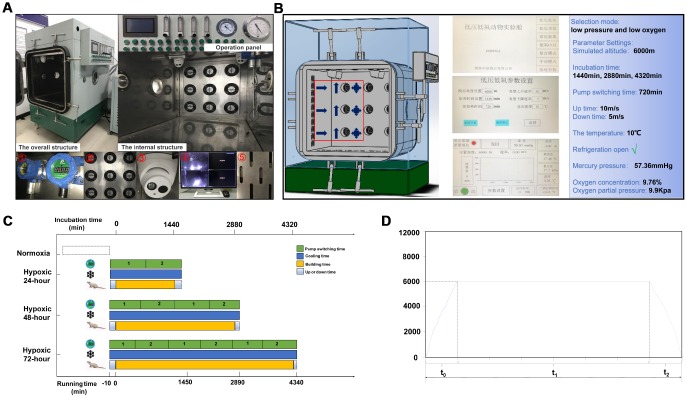
**Experimental hypoxia chamber.** (**A**) The hypoxia chamber for animal experiments includes an external operation panel and an internal cabin; ① vacuum pump; ② fans; ③ monitor device; ④ monitor screen; ⑤ cooling hole. (**B**) Parameter settings. The red box indicates cabin’s air flow setup: cold air enters, air is discharged, and air is exchanged. (**C**) Animal modeling time and equipment running process. (**D**) Time-height curve, comprising a rising stage (t0), cultivated room running time (t1), and a constant speed drop phase (t2).

The setup of the oxygen chamber (DS850-I, Weifang Huaxin Oxygen Industry Co., Ltd.) is shown in Figure 10A. The chamber was programmed to operate on low pressure and low oxygen mode, with the cooling feature turned on. Through the operation interface, parameters such as altitude, incubation time, pump switching time, altitude rise/fall rate, and temperature were adjusted. Real-time monitoring was performed during equipment operation (Figure 10B).

### Hypoxia modeling setup

After placing the rats in the incubation chamber inside the cabin platform the device was powered on and the "low pressure and low oxygen" mode was selected. The parameters were set to: simulated height, 6,000 m; incubation times, 1,440 min, 2,880 min, and 4,320 min; pump switching time, 720 min. Height rising rate was 10 m/s, height falling rate was 5 m/s, and temperature was set to 10°C. The “cooling on” mode was also set, and the operation of the device was initiated. After reaching the preset height, incubation chamber timing started and the modeling process begun. At this time, pressure was 56.30 mmHg, O_2_ concentration was 9.76%, and O_2_ partial pressure was 9.9 kPa. During the modeling period, the rats had free access to water and food. After the modeling was completed, the air pump stopped working and the device reset automatically. At this time, the switching mode was set to "low oxygen carbon dioxide" to ensure that the O_2_ concentration in the cabin continued to be maintained at about 9.76% until the height dropped to 0 m (Figure 10C and 10D).

### Hypoxic cell model

Pulmonary microvascular endothelial cells (PMVECs) were purchased from Cyagen Biosciences (Guangzhou, China) and grown in M199 medium (Cyagen Biosciences) supplemented with 10% heat-inactivated fetal bovine serum (FBS) (Cyagen) and endothelial growth factor (EGFS) at 37°C under 5% CO_2_ and 2% O_2_. Hypoxia incubators (HF100, Heal Forcer) were used to culture PMVECs under hypoxic conditions for 0, 24, 48, and 72 h, with media changes every other day.

### Measurement of arterial blood gasses

Oxygen partial pressure (PaO_2_), carbon dioxide partial pressure (PaCO_2_), oxygen saturation (SaO_2_), potential of hydrogen potential of hydrogen(PH), plasma bicarbonate (HCO_3_) concentration, plasma total carbon dioxide (TCO_2_) concentration, and base excess extracellular fluid (BEecf) were analyzed in arterial blood samples using an iSTAT-200 Portable Clinical Blood Gas Analyzer (Abbott, Illinois, USA).

### Total BALF protein content and lung W/D determinations

Total BALF protein content was assessed using a BCA protein concentration assay kit (Solarbio, Beijing, China) by interpolation into a standard calibration curve. Absorbance at 562 nm was measured using an ultra-micro UV spectrophotometer (IMPLEN, NanoPhotometer NP80 Mobile). W/D was recorded using an electronic scale (Mettler Toledo ME403E). Wet weight was first measured after excess fluid (blood and water) removal. Subsequently, dry weight was determined after drying the lung tissue at 60°C for 48 h.

### Histological analyses and immunohistochemistry

Rats’ right middle lung lobes were isolated and fixed in 4% paraformaldehyde for 24 h. Five μm thick paraffin sections where processed and stained with H&E and evaluated under an optical microscope (DMI3000B; Leica, Wetzlar Germany). Antigen retrieval for immunohistochemistry was carried out after dewaxing and hydration of the sections by heating in 500 ml of 10% citric acid buffer (pH 6.0) for 10 min. An endogenous peroxidase blocker (ZSGB-BIO, Beijing, China) was next added for 10 min at room temperature, following by incubation with normal goat serum working solution (ZSGB-BIO, Beijing, China) for 10-15 min at room temperature. Then, antibodies against occludin(1:200, rabbit lgG; Abcam, USA), notch1(1:400, rabbit lgG; Proteintech Group, Inc., Chicago, IL), CD31(1:2000, rabbit lgG; Abcam, USA), Hes-1(1:200, rabbit lgG; Abcam, USA), VEGF-A (1:200, mouse lgG; Abcam, USA) and VEGF-R2 (1:250, rabbit lgG; Abcam, USA) were applied and incubated at 4°C overnight. Biotinylated goat anti-rabbit/mouse IgG (ZSGB-BIO, Beijing, China) and HRP-conjugated streptavidin/avidin antibody working solutions (ZSGB-BIO, Beijing, China) were added with 10-15 min incubations at room temperature. Freshly prepared DAB solution (Solarbio, Beijing, China) was used for signal detection, followed by counterstaining with hematoxylin.

### RNA isolation and miRNA microarray analysis

RNA was extracted from lung tissue samples using the mirVana^TM^ PARIS^TM^ Serum/Plasma Kit (Cat# AM1556, Ambion, Austin, TX, US). RNA quantity was determined by a NanoDrop ND-2000 device (ThermoFisher, Boston, MA, USA) starting with 1 μg total RNA, and RNA quality was measured with an Agilent 2100 Bioanalyzer (G2939A; Agilent Technologies, Santa Clara, CA, USA). After poly(A) tailing of the total RNA, the FlashTag Biotin HSR Ligation Mix (P/N 901911, Affymetrix) was added into the tailed mixture for biotin labeling and probe purification in accordance with the manufacturer’s instructions. A GeneChip Hybridization Oven 645 (Affymetrix, Santa Clara, CA, US) was employed to hybridize the samples with the Affymetrix miRNAs 4.0 microarray (Biotechnology co. LTD, Beijing, China), followed by chip elution using the Fluidics Station 450 (Affymetrix, Santa Clara, CA, US). A GeneChip Scanner 30007G (Affymetrix, Santa Clara, CA, US) was used for array scanning. Raw data were analyzed using GCOS software (Affymetrix, Santa Clara, CA, US), and differential expression was determined for genes with p < 0.05 and FC > 1.5.

### qRT-PCR

Total RNA was extracted from lung tissues and cells using TRIzol reagent (Solarbio, Beijing, China) following the manufacturer’s protocol. The extracted RNA was quantified by measuring absorbance at 260 nm, followed by reverse transcription reaction using the PrimeScriptTM RT Reagent Kit (RR037A; TAKARA, JAPAN). Real-time PCR was carried out using a real-time PCR (Bio-RAD) reaction system (Table 4). After the reaction, melting curve analysis was performed, and the data collected was normalized to U6 snRNA expression data. Relative gene expression levels were quantified based on the 2^-ΔΔCt^ method.

**Table 4:** Primers of miRNA used for qRT-PCR in this study.

**Forward primer**	**Sequence (5′to3′)**
rno-miR-203a-3p	TGCGGGTGAAATGTTTAGGACCAC
rno-miR-30b-5p	TGCGGTGTAAACATCCTACACTCA
rno-miR-532-5p	TGCGGCATGCCTTGAGTGTAGGAC
rno-miR-16-3p	TGCGGACCAATATTATTGTGCTGC
rno-miR-101a-5p	TGCGGTCAGTTATCACAGTGCTGA
rno-miR-375-3p	GAAGATCTTGAGTACAGGGGCCAG
VEGF-R2	CTCCATCTTTTGGTGGGATG
VEGF-A	CGACAGAAGGGGAGCAGAAAG
Notch1	CTGAGGCAAGGATTGGAGTC
Hes-1	GCCAGTGTCAACACGACACCGG

### Western blotting

Frozen lungs’ right lower lobes were weighed, and 1 ml RIPA buffer was added to each 50-100 mg tissue fragments. Protein lysates were extracted from cells. Subsequently, magnetic beads (Thermo Fisher Scientific Inc., USA) were added, and the mix was homogenized through a high-flux tissue grinder and centrifuged at 12,000 g/min for 15-30 min to collect the supernatant. Following protein concentration determination (BCA protein concentration assay kit; Solarbio, Beijing, China), protein extracts from tissue and cell culture samples were lysed with 1x loading buffer, boiled for 5 min, and subjected to SDS-PAGE. Proteins were next transferred onto PVDF membranes (LIUYI, Beijing, China) at 4°C applying 200 mA (constant current) for 1 h. The membranes were then immersed in blocking solution for 30 min at room temperature, cut at the imprinted position of the protein, and incubated with notch1(1:1000, rabbit lgG; Proteintech Group, Inc., Chicago, IL), Hes-1(1:200, rabbit IgG; Abcam, USA), VEGF-A (1:200, mouse lgG; Abcam, USA), VEGF-R2 (1:250, rabbit lgG; Abcam, USA), GAPDH (1:5000, Proteintech Group, Inc., Chicago, IL) and Tubulin (1:1000, Abcam, USA) for 2 h at room temperature. Afterwards, the membranes were rinsed with TBST and incubated with corresponding secondary antibodies (1:3000, HRP-conjugated goat anti-rabbit/mouse IgGs; Proteintech Group, Inc., Chicago, IL) in TBST at room temperature for 1 h. After rinsing with TBST, the membranes were developed using Super ECL Plus luminescent solution for 2 min and fixed.

### Transduction and transfection experiments

PMVECs were cultured overnight and transduced with HBLV-GFP-PURO (negative control, NC), HBLV-miR-203a-GFP-PURO (miR-203a-3p-mimics), HBLV-VEGF-shRNA-GEP-PURO (VEGF-shRNA) vectors. PMVECs that transduced with HBLV-VEGF-shRNA-GFP-PURO vectors simultaneously transfected with antisense oligonucleotides-negative control (VEGF-shRNA+ASO-NC) or antisense oligonucleotides (VEGF-shRNA+ASO). After 24 hours, fresh medium was added and the culture was continued. Infection efficiency was analyzed by assessing cell fluorescence at 72 h.

### Enzyme-linked immunosorbent assay (ELISA)

IL-6, TNF-α, and IL-10 expression in lung tissues and PMVECs was analyzed using ELISA kit (Nanjing Jiancheng Bioengineering Institute, Nanjing, China) according to the protocols’ instructions.

### Apoptosis assay

PMVECs were cultured in 24-well plates and infected with lentiviral vectors the next day. After 48 hours, cells were incubated under normoxia or hypoxia for 24, 48, or 72 h. Cells were next harvested, washed twice with PBS, and re-suspended in 100 μl of binding buffer plus 5 μl of Annexin V/FITC (Solarbio, Beijing, China). The cells were incubated at room temperature in the dark for 5 min, and 10 μl of propidium iodide (PI; 20 μg/ml) plus 400 μl PBS were added for immediate flow cytometry analysis.

### Angiogenesis assay

Matrigel was melted overnight at 4°C and diluted 1:1 with no-additive, M199 base medium. The solution was evenly spread in 96-well plates (50 μL/well) and incubated at 37°C for 30 min to allow gelation. Hypoxia-treated PMVECs were harvested into 2×10^5^ cells/mL suspensions. A 100 μL cell suspension aliquot was added onto the Matrigel surface on each well and incubated for 4 h. The vascular network structure was observed under a microscope, and the number of vascular rings was determined.

### Cell viability assay

PMVECs (3X10^4^ cells/well) were seeded into 24-well plates and infected with viral vectors 24 h later. After 4 h/days, the cells were harvested and 1X10^4^ cells/well were placed in 96-well plates for hypoxic treatment. After 0, 24, 48 and 72 h, 10 μl of CCK-8 detection solution was added to each well and incubated at 37°C for 2 h. Absorbance was measured at 450 nm on a microplate reader (BIOBASE-EL10A). Death rate was calculated as (absorbance of NC control - absorbance of experimental group)/absorbance of control group.

### Dual-luciferase reporter assay

PMVECs cells were transfected with reporter pmirGLO plasmid vectors to establish the following cell groups: mimics-NC (negative control), miRNA-203a-3p-mimics, VEGF-UTR+mimics-NC (wild-type VEGF 3′-UTR), VEGF-mUTR+mimics-NC, (mutant VEGF 3′-UTR), and VEGF-mUTR+miRNA-203a-3p-mimics. A Dual-Luciferase Reporter Gene Assay Kit (Promega, Shanghai, China) was used to assess the expression of miR-203a-3p and VEGF-A following the kit instructions. Sequences are listed in Table 5.

**Table 5:** The sequence of miRNA mimics, mimics-NC, ASO, and VEGF used in this study.

**Name**	**Sequence (5′to3′)**
miR-203a-3p-mimics-NC (mimics negative control)	UCACAACCUCCUAGAAAGAGUAGA
miR-203a-3p-mimics	CUAGUGGUCCUAAACAUUUCACTT
miR-203a-3p+ASO	GUGAAAUGUUUAGGACCACUAG
VEGF-TOP	CTTGGTTAATATTTA ATTTCAAC
VEGF-mTOP	CTTGGTTAATATTTA TAAAGTTC

### Statistical analysis

SPSS 20.0 was used for all statistical analyses. Data are expressed as mean ± standard error of the mean (SEM). Comparisons between two groups were performed using independent samples t-test. For three or more groups, differences were compared through one-way analysis of variance (ANOVA) and Fisher’s Least Significant Difference (LSD) test. Dunnett’s T3 test was applied in the presence of variance heterogeneity. P < 0.05 was considered significant.
